# Primary Intracranial Ewing Sarcoma/Peripheral Primitive Neuroectodermal Tumor Mimicking Meningioma: A Case Report and Literature Review

**DOI:** 10.3389/fonc.2020.528073

**Published:** 2020-10-06

**Authors:** Yining Jiang, Liyan Zhao, Yubo Wang, Xinrui Liu, Xinmin Wu, Yunqian Li

**Affiliations:** ^1^Department of Neurosurgery, First Hospital of Jilin University, Changchun, China; ^2^Department of Medical Laboratory, Second Hospital, Jilin University, Changchun, China

**Keywords:** ewing sarcoma, peripheral primitive neuroectodermal tumor, primary intracranial, diagnosis, central nervous system, treatment

## Abstract

**Background:** Primary intracranial Ewing sarcoma (ES)/peripheral primitive neuroectodermal tumors (pPNETs) are extremely rare malignancies, which arise in children and adolescents, with only 9 cases reported in patients over 30 years of age. Due to its rarity, MRI features and treatment strategies for primary intracranial ES/pPNETs remain unclear. The purpose of this study was to explore the clinical features, imaging findings, pathological characteristics, different diagnoses, treatment, and prognosis of cerebellar liponeurocytoma in adults.

**Case Description:** A 55-year-old female was admitted to the hospital with memory decline over 1 month, which aggravated in the last 2 weeks. MRI showed a 4.3 × 6.5 × 3.5 cm heterogeneous large mass in the left frontal lobe with mild peritumoral edema. The mass was successfully removed under neuronavigation and electrophysiological monitoring. The entire mass was removed, and postoperative pathology indicated an ES pPNET diagnosis, with an *EWSR1* gene rearrangement. Subsequently, the patient underwent disciplinary radiotherapy.

**Conclusion:** The diagnosis of primary intracranial ES/pPNETs depends on the comprehensive consideration of histological examination, immunohistochemical analysis, and genetic detection. Gross tumor resection combined with radiotherapy and chemotherapy might be the most beneficial treatment.

## Introduction

Primary intracranial ES/pPNETs are a group of poorly differentiated, highly malignant, and aggressive small round cell neoplasms, generally originating from bone and soft tissue, which are common among children and adolescents (1–4). ES/pPNETs that occur intracranially are extremely rare, with only 57 cases reported so far. For patients over 30 years of age, only 9 cases have been reported (4–12). Fusion of the *EWSR1* gene with a member of the ETS gene family is thought to be the primary cause of the ES/pPNETs (3, 4, 13–15). Herein, we present a case of ES/pPNET located in the left frontal lobe of a 55-year-old female, who was tested through histological examination, immunohistochemical analysis and fluorescence *in situ* hybridization (FISH). Moreover, we have concluded the patients over 30 years of age, and summarized the typical pathological and radiological features of this rare tumor entity, with surgery, adjuvant therapy, prognosis, and important differential diagnoses discussed in detail.

## Case Report

### History and Examination

A 55-year-old female patient was admitted to the hospital due to progressive memory decline for over 1 month, which aggravated in the last 2 weeks. Physical examination showed normal higher mental functions. Left limb muscle strength was normal; right limbs were slightly weaker than left limbs.

### Neuroimaging Finding

**Figure 1:**
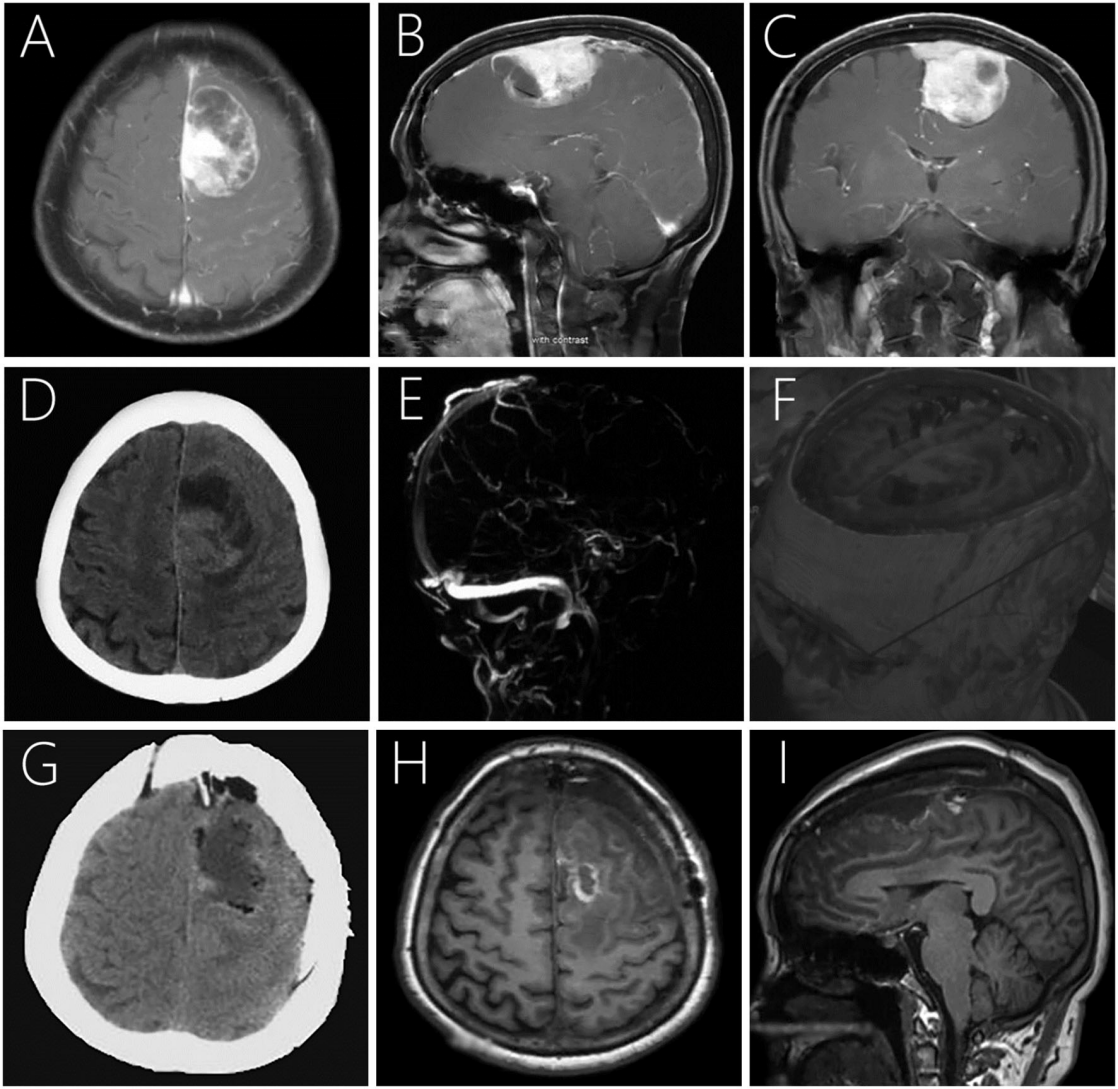
Post-contrast MRI **(A–C)** showing a large irregular mass with heterogeneous enhancement in the left frontal lobe with mild peritumoral edema. Plain CT demonstrating a large mass mixed hypo- and isodensity **(D)**. Magnetic resonance venography showing no development was seen in the superior sagittal sinus of the frontal area **(E)**. Diffusion tensor imaging showing compression, displacement, and partial interruption of nerve fibers in the lesion area **(F)**. Postoperative CT **(G)** and MRI 7-days postoperatively **(H,I)** revealed that the lesion was completely removed with no signs of recurrence.

Magnetic resonance imaging (MRI) showed a 4.3 × 6.5 × 3.5 cm large irregular mass located in the left frontal lobe with mixed isointense-to-hypointense signals on T1-weighted imaging (T1WI), uneven hypointense-to-hyperintense signals on T2-weighted imaging (T2WI), and T2 dark-fluid. After gadolinium administration, obvious heterogenous enhancement was observed (Figures 1A–C). Computerized tomography (CT) showed a heterogeneous hypo-and isoindense mass in the left frontal lobe with a CT value of 15–37 HU (Figure 1D). Magnetic resonance venography indicated that the forehead sagittal sinus was not visible as it was compressed by the tumor (Figure 1E). Diffusion tensor imaging showed that the nerve fibers in the lesion area were compressed, displaced, and partially interrupted (Figure 1F).

### Surgery

A preoperative clinical diagnosis of meningioma was made, and surgery was performed under preoperative neuronavigation and intraoperative electrophysiological monitoring of somatosensory, and muscle-evoked potentials. The tumor mass was soft, rich in blood supply, and invaded into the sagittal sinus from the left side. The tumor and eroded dura were completely removed.

### Pathological and Genetic Finding

**Figure 2:**
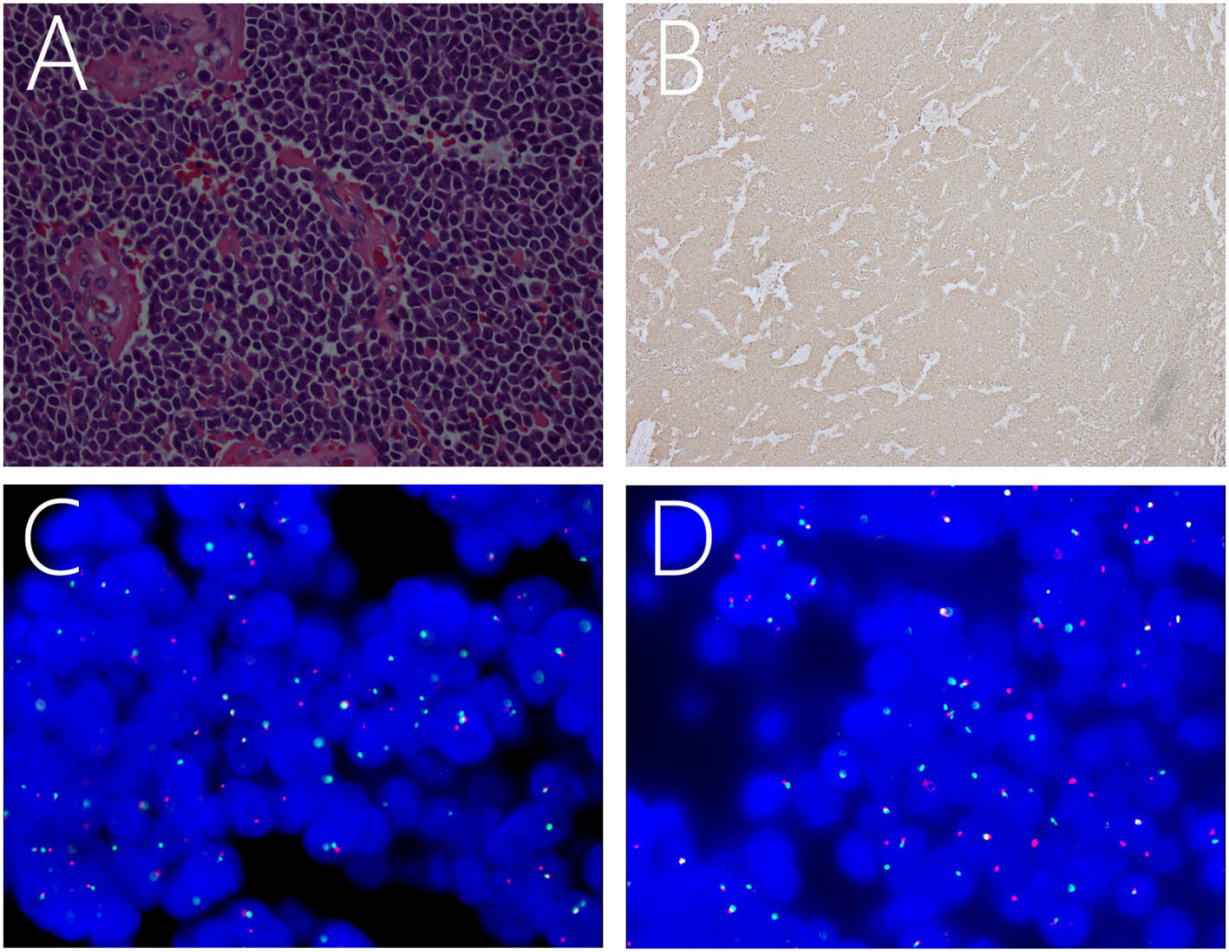
Light microscopic image showing a number of small, round, oval, undifferentiated cells, with intense staining of the nuclei and basophilic cytoplasm **(A)**. Immunohistochemical staining showing positivity for CD99 **(B)**. FISH revealing positive results for EWSR1 rearrangement as indicated by the separation of red and green signals **(C,D)**.

Pathology was suggestive of ES/pPNET. Hematoxylin and eosin-stained paraffin sections predominantly showed closely packed small, round to oval, undifferentiated cells with hyperchromatic nuclei, increased mitotic activity, and little basophilic cytoplasm (Figure 2A). Immunohistochemical analysis indicated that the Ki-67 index was ± 50%. Moreover, the neoplasm was positive for CD99 (Figure 2B), FLI-1, MAP-2, vimentin, synaptophysin, and the progesterone receptor, and was negative for TTF-1, CK-pan, CK5/6, CD56, CgA, LCA, S-100, glial fibrillary acidic protein, myeloperoxidase, MyoD1, NeuN, Olig-2, STAT6, and epithelial membrane antigen. Moreover, fluorescence *in situ* hybridization (FISH) with a *EWSR1* break apart probe showed 41.0% of split signals, thereby confirming the diagnosis of ES/pPNETs (Figures 2C,D).

### Postoperative Course

The patient's postoperative course was uneventful, and positron emission tomography was performed to identify potential extracranial primary sites, and serum tumor markers were measured, but both were negative. Postoperative CT (Figure 1G) and MRI 1 week postoperatively (Figures 1H,I) demonstrated that the lesion was completely removed, and no signs of recurrence were observed. Subsequently, the patient underwent disciplinary radiotherapy for 1 month. During the last telephone follow-up in August 2020, 18 months after surgery, the patient reported to be living a normal daily life with no apparent symptoms. We believe the patient's condition is stable and will continue to follow-up.

## Discussion

### Epidemiology

Intracranial ES/pPNETs are very rare malignant tumors, accounting for 0.03% of the total number of intracranial tumors (3, 4), with only 57 cases reported until now. ES/pPNETs mostly occur in children and adolescents with a median age at first onset of disease being 15 years of age, and the peak of disease is prominent in the second decade, ranging from 5 months to 67 years of age, with a slight male predisposition (14, 16–18). The case presented here is an ES/pPNET in the left frontal lobe of a 55-year-old female patient. Notably, the incidence of ES/pPNET in adults is exceedingly rare. Here, we reviewed all reported cases in patients older than 30 years of age, only 9 cases have been reported. Our study is the first to report a systematic review for primary intracranial ES/pPNET in adult patients. In our study, the mean patient age was 50.7 years (range: 34–67 years), and there were seven females and four males (Table 1). Moreover, supratentorial cerebral hemispheres are predilection sites for ES/pPNET (14). In our study, the presenting sites include the hemispheric surface (6 cases), cerebellopontine angle (2 cases), cavernous sinus (1 case), and posterior fossa (1 case). Moreover, the origin of primary intracranial ES/pPNETs has not been clearly elucidated, however, the presumptive precursor cells are speculated to stem from the neural crest or mesenchymal stem cells (19, 20).

**Table 1:** Summary of Cases of Primary Intracranial ES/ pPNETs over 30 years of age Reported in the Literature.

**Author reference/** **year**	**Age(y)** **/sex**	**Onset** **of** **symptoms** **(months)**	**Main** **symptoms** **and** **signs**	**Location**	**Dural based**	**T1WI**	**T2WI**	**Enhance** **-ment** **contrast**	**Tumor** **great** **diameter** **(cm)**	**Bone involve** **-ment**	**Sugery**	**Chemotherapy**	**Radio** **-therapy**	**Recurrence**	**Postsurgical Metastasis**	**Follows-up**
Present case	55/F	1	Memory decline, muscle strength decrease	Left frontal lobe	Y	Hetero -geneous	Hetero -geneous	Hetero -geneous	6.5	Slight	GTR	None	55 Gy	None	None	Stable disease for 18 months.
Chen et al. (4) 2019	43/M	1	Epilepsy	Right parietal lobe	Y	N/A	N/A	Hetero -geneous	N/A	None	GTR	VAC+ actinomycin D	50 Gy	Y	None	Died after 48 months.
Vanden -Heuvel et al. (5)/2015	61/M	2	Left hemiparesis, left-side facial drooping, aggressive fatigue, and weakness	Right frontal and temporal lobe	Y	N/A	N/A	Heterogeneous	6.2 5.1 cm	None	Biopsy	None	None	N	None	Lost contact after first follow-up.
Salunke et al. (6)/2014	52/M	1	Low-grade fever, headache, vision diminution, ataxia, and occipital mass swelling	Posterior fossa	Y	N/A	N/A	Hetero -geneous	6 cm	Y	STR	Two cycles of VAC (alternating with EI every 3 weeks) for 4 months.	50 Gy	Y	None	Recurrence 6 months later and died in 2 weeks.
Cole et al. (7)/2014	51/F	N/A	Visual disturbances	Occipital parafalcine region	Y	N/A	N/A	Hetero -geneous	3.5 3 cm	None	GTR	VCD (alternated with EI every 3 weeks) for 14 cycles. After the fourth cycle, doxorubicin was displaced into dactinomycin.	None	N	None	Monitored every 3 months, with no evidence of recurrence for 24 months.
Mellai et al. (8)/2010	56/F	N/A	Headache, confusion and a left-side hemiparesis	Right temporal region	Y	Hypo	Hyper	Heterogeneous	N/A	None	GTR	N	None	None	None	Asymptomatic for 18 months after surgery.
Attabib et al. (9)/2006	48/F	6	Headache, left ptosis, left periorbital, and maxillary region numbness	Left cavernous sinus	Y	N/A	N/A	Intense, diffuse	4.0 cm	None	Partial removal	VCD (alternated with EI every 3 weeks)	54 Gy	None	None	Stable disease for 14 months.
D'Antonio et al. (10)/2004	50/F	Several	Progressively severe headaches, vomiting, and drowsiness	Right parieto-temporal region	Y	Iso	Hypo	Intense, diffuse	6.0 cm	None	GTR	None	None	None	None	Disease free at 12 months.
Kalamarides et al. (12)/2001	34/F	12	Vertigo, left pulsatile tinnitus, gait disturbance	Left CPA	N	N/A	Hetero -geneous	Intense, diffuse	18 mm	None	STR	None	Craniospinal radiotherapy (35 Gy with an overdosage to 55 Gy over the tumor bed)	Y	None	No recurrence was found in 12-month follow-up.
Simmons et al. (11)/2001	67/F	18	Right facial pain, facial palsy, hearing disturbance, headache	Right CPA	N	N/A	N/A	Hetero -geneous	N/A	None	Biopsy	None	Y	Y	Y	No symptoms progression for 13 months, followed by rapid decline and death.

*CPA, cerebellopontine angle; GTR, gross total resection; STR, subtotal resection; N/A, not available; V, vincristine; A, adriamycin; C, cyclophosphamide; E, etoposide; I, ifosfamide; D, doxorubicin*.

### Clinical Presentation

The clinical manifestations of primary intracranial ES/pPNETs are diverse and highly variable, considering tumor location, size, and invasion site. Our study showed that clinical symptoms include intracranial pressure, increasing presenting headache, and vomiting (5 cases, [50.0%]), hemiplegia and muscle strength decrease (3 cases, [30.0%]), facial palsy (3 cases, [30.0%]), deafness and hearing disturbance (2 cases, [20.0%]), drowsiness and fatigue (2 cases, [20.0%]), epilepsy (1 case, [10.0%]), memory decline (1 case, [10.0%]), and ataxia (1 case, [10.0%]). The mean duration of clinical symptoms was 5.9 months (range: 1–18 months), and the duration in our results is longer than what has been described in the literature (with a median of 9 weeks) (14). This might associate with the compensation of unclosed or not firmly integrated cranial sutures in children. Some highly uncommon clinical signs of ES/pPNETs have been reported, including the formation of large intra-cerebral hematomas and elevated serum levels of carcinoembryonic antigens (1), which are important to be noticed during clinical evaluation. In this case, the patient's major complaint was memory decline, suggesting functional damage of the dominant side frontal lobe.

### Radiologic Features

We extensively reviewed previous reports and found that primary intracranial ES/pPNETs mostly showed mixed isointense-to-hypointense signals on T1WI, and isointense-to-hyperintense signals on T2WI (4). Our case was consistent with these findings. As for post-contrast MRI, Cherif et al. reviewed all reported ES/pPNETs in 2018, and presented a heterogeneous enhancement of about 40.0%, intense enhancement of about 52.5%, and moderate enhancement of 7.5% of these lesions (14). In our study, we showed heterogeneous enhancement of about 60.0% (6 cases), and intense enhancement of 40.0% (4 cases) of these lesions. Jing et al. regarded the reason for such heterogeneous enhancement as a characteristic of high density small round cells under the microscope, and a large amount of protein-rich mucus in some areas, which was accompanied by hemorrhage and necrosis (16). In our study, skull involvement was observed in two cases. In addition, tumors with a dural base were another characteristic of primary intracranial ES/pPNET, which was observed in 80.0% in patients included in our study. This was one of the key aspects for ES/pPNET to be confused with hemangiopericytoma or meningioma among others (5, 21). In our case, the tumor adhered to the sagittal sinus with a broad base and grew in a semi-circular shape, with a preoperative diagnosis of hemangiopericytoma. This was probably due to the ES/pPNET undermining surrounding normal meninges. Furthermore, the meninges, blood-brain barrier, and skull affected the growth pattern of the tumor. Therefore, to some degree, imaging in general and MRI examinations have limited value in the differential diagnoses of ES/pPNET (22).

### Diagnosis

ES/pPNETs are highly aggressive, malignant tumors with focal necrosis (1). They are mainly composed of small, round or oval, undifferentiated cells, with hyperchromatic nuclei, increased mitotic activity, and a slightly basophilic cytoplasm (1, 3, 4). Moreover, the tumor cells are markedly fibrotic, highly mitotic, and separated by groups of cells containing collagen bands (1, 3, 4, 14, 18). Membranous expression of CD99 is a highly reliable and sensitive diagnostic biomarker for primary intracranial ES/pPNETs (3, 4, 13–15, 18), and was detected in nearly all patients. However, CD99 is not recommended as a specific immunohistochemical marker for diagnosing ES/pPNETs, because CD99 can also be detected in other small, blue round cell tumors, including lymphoblastic lymphomas, ependymomas, and rhabdomyosarcomas (2, 4, 14, 21, 23). Nevertheless, the staining pattern in these cases is often cytoplasmic, rather than the distinct membranous staining typical of ES/pPNETs (1, 17, 22, 24). In general, the membrane protein FLI-1 is also expressed in ES/pPNETs (1, 4).

At present, molecular testing depicting *EWSR1* gene rearrangement is a golden standard for diagnosing ES/pPNET (13, 14), which can be detected by reverse transcription polymerase chain reaction and FISH methods with a sensitivity and specificity about 91–100% (5). Chromosomal translocation *t*(11, 22)(q24;q12) is the most common genetic aberration in ES/pPNETs. This translocation results in formation of a chimeric transcription factor *EWS-FLI-1* (friend leukemia integration 1 transcription factor) in 85–90% of ES/pPNETs, being the in-frame fusion of the 5' end of *EWSR1* gene with the 3' portion of the FLI-1 gene with abnormal transcription regulator properties (1, 4, 13–15, 18). The downstream effects of the *EWS-FLI-1* fusion gene include dysregulation of cell proliferation, differentiation, apoptosis, angiogenesis, invasion, and metastasis (25). There are also other chromosomal translocations, including *ESW/ERG t*(21, 22)(q22;q12), which is the second most common translocation, accounting 10% of ES/pPNETs (18). Less than 1% of chromosome translocations lead to *EWSR1* gene fusion with other ETS family transcription factor genes, including *t*(2, 22) (q36;q12) (*EWS-FEV*), *t*(7, 22) (q22;q12) (*EWS-ETV1*), *t*(17, 22) (q21;q12) (*EWS-EIAF*) (1, 26–28). Nonetheless, it is worth noting that these translocations are not only associated with ES/pPNETs. Thorner et al. demonstrated that two rhabdomyosarcomas and two polyphenotypic tumors with *t*(11, 22)(q24;q12) translocations were negative for CD99 (29). Thus, the diagnosis of ES/pPNETs is highly dependent on the comprehensive consideration of histological examination, immunohistochemical analysis, and molecular genetic analyses (18). The pseudoautosomal gene, *MIC2* gene, is another important gene, which has been demonstrated to be present in ES/pPNETs (17, 18). The cell surface glycoprotein CD99 is the product of the *MIC2* gene, which is observed in essentially all cases of ES/pPNETs (15, 17).

In differential diagnosis, due to histological similarities, primary intracranial ES/pPNETs can be misdiagnosed as central nervous system embryonal tumors, cPNETs (such as medulloblastoma, central neuroblastoma, and other neuroepithelial tumors), malignant meningioma, and melanoma, among others (3, 18, 20, 30). Central nervous system embryonal tumors do not express the *MIC2* gene, and are negative for CD99 (13). As for cPNETs, they can be confirmed by the absence of CD99 expression and *t*(11, 22) translocation (1, 2, 14, 22). In our clinical case, diffuse membranous positivity for CD99 and FLI-1 were observed, combined with the expression of *EWSR1* in FISH. Furthermore, whole-body CT, bone marrow aspirates, bone scans, lumbar punctures, and positron emission tomography scans are recommended to confirm the tumor to be primarily intracranial.

### Treatment Modalities

Due to the rarity of ES/pPNETs, the standard treatment approach for this type of malignancy has not yet been established. To date, surgical resection, with the aim of total tumor resection, is the main therapy cornerstone (3, 4). Wide surgical resection margins at the time of primary surgery have markedly reduced local recurrences (13, 15). Chen et al. found the lifetime of gross total resection (GTR) to be significantly longer when compared to partial resection (38 months vs. 20 months, respectively) (4). In our study cohort, GTR was performed in five out ten patients, subtotal resection two patients, biopsy in two patients, and partial excision in one patient. The mean follow-up period for patients with GTR was 24 months (range: 12–48 months), with 1-year follow-up for 100.0% (5/5), 1.5-year for 80.0% (4/5), and 2-year for 40.0% (2/5). However, the mean follow-up period for patients with incomplete tumor resection (ITR) was 11.3 months (range: 6–14 months), with 1-year follow-up for 60.0% (3/5) and 1.5-year follow-up for none.

Due to the rarity of this disease, the standard first-line adjuvant treatment remains unclear. Some studies showed that the use of chemotherapy significantly improved the long-term survival rate, from 5–10 to 70–80% (31, 32), and presented lower recurrence rates (4, 14, 31, 32). In our study cohort, no recurrence was found in patients who underwent ITR and chemotherapy. However, for three patients who only underwent ITR, recurrence was found in two patients. Moreover, the mean follow-up period for patients with chemotherapy (23 months) was longer than for patients who underwent surgery only (11 months). The chemotherapeutics included vincristine, cyclophosphamide, doxorubicin, ifosfamide, etoposide, Adriamycin, and actinomycin D (3, 14). The chemotherapeutic combinations in our study include vincristine-cyclophosphamide-doxorubicin (VCD), vincristine-adriamycin-cyclophosphamide (VAC), and VAC-actinomycin D (VACA) alternating with etoposide-ifosfamide (EI), which was consistent with the findings presented in previous studies (4, 14). Moreover, the European Cooperative Group proposed vincristine-ifosfamide-doxorubicin-etoposide (VIDE) as the intensive induction chemotherapy for Ewing sarcoma (33). In addition, Jain et al. proposed neoadjuvant chemotherapy, which improved cytoreduction and achieved local control of tumors prior to surgical resection (4, 34). Moreover, radiotherapy is known to be an important adjuvant treatment option for ES/pPNETs (14). Chen et al. (4) suggested that the 1- and 2-year survival rates of patients receiving adjuvant radiotherapy (88.9 and 66.7%) were significantly higher than those of patients who did not receive radiotherapy (60.0 and 0%). In addition, the median survival time was significantly extended (38 months vs. 13 months). However, in our study, due to the small sample size, there was no significant survival discrepancy in patients with or without radiotherapy.

After extensive literature review, we observed that GTR combined with adjuvant chemotherapy and radiotherapy was the most beneficial treatment protocol (3, 4, 14, 21). Moreover, close follow-up is recommended and essentially necessary. Chen et al. presented that patients who underwent GTR, chemotherapy, and radiotherapy had a longest 2-year survival rate and the longest median survival time (4). In our study cohort, only one patient underwent GTR, adjuvant chemotherapy and radiotherapy, and he showed the longest survival period of 4 years. Notably, for patients who underwent ITR, radiotherapy combined with chemotherapy is highly recommended (3, 14).

### Prognosis

Primary intracranial ES/ pPNETs are aggressive malignant tumors with a poor prognosis, and ITR is one of the most important reasons for its recurrence (14). Therefore, GTR should be the surgical aim. However, even when treated with total resection, recurrence may be unavoidable. Moreover, postoperative metastasis along cerebrospinal fluid was observed in one case in our study (11). Tumors located in the infratentorial region, with skull involvement, metastasis, and postoperative radio- and chemotherapy are highly associated with a poor prognosis (14). Adversely, age, sex, onset of symptoms, and presence of hemorrhagic or cystic component, do not appear to influence the prognosis of ES/pPNETs (14, 18, 33).

Furthermore, with an emerging understanding of the molecular underpinnings of ES, a prognosis based on the biological profile of tumors is possible. For example, a better prognosis has been associated with tumors containing the *EWS-FLI-1* chimeric-type gene, when compared with other types of gene translocation (35).

## Conclusion

In summary, primary intracranial ES/pPNETs are rarely reported in the literature and, with only 9 known cases, is extremely rare in patients over 30 years of age. Thus, more cases describing ES/pPNETs as well as long-term follow-up studies are warranted to fully understand ES/pPNETs in the adult population. Therefore, because of these limitations, our present case report might represent an additional reference among the few available that might serve as a potential guide for clinicians and radiologists.

## Data Availability Statement

All datasets generated for this study are included in the article/supplementary material.

## Ethics Statement

The studies involving human participants were reviewed and approved by the First Hospital of Jilin University Ethics Committee. The patients/participants provided their written informed consent to participate in this study. Written informed consent was obtained from the individual(s) for the publication of any potentially identifiable images or data included in this article.

## Author Contributions

YJ, LZ, and YW made study design, data collection, data analysis and interpretation, and composed the manuscript and literature review. YL and XL was the surgeon that performed the surgery and did data collection, data analysis, and interpretation. XW made English and grammar corrections, critical revisions, and approved final version. YL had the acquisition, analysis or interpretation of data for the work, revising it critically for important intellectual content, final approval of the version to be published, and agreement to be accountable for all aspects of the work in ensuring that questions related to the accuracy, or integrity of any part of the work are appropriately investigated and resolved. All authors contributed to the article and approved the submitted version.

## Conflict of Interest

The authors declare that the research was conducted in the absence of any commercial or financial relationships that could be construed as a potential conflict of interest.

## Abbreviations

CT: Computerized tomography
ES/pPNETs: Ewing sarcoma/ peripheral primitive neuroectodermal tumors
FISH: Fluorescence *in situ* hybridization
GTR: Gross total resection
ITR: Incomplete tumor resection
MRI: Magnetic resonance imaging
T1WI: T1-weighted imaging
T2WI: T2-weighted imaging.
